# Targeting Neuroplasticity, Cardiovascular, and Cognitive-Associated Genomic Variants in Familial Alzheimer’s Disease

**DOI:** 10.1007/s12035-018-1298-z

**Published:** 2018-08-15

**Authors:** Jorge I. Vélez, Francisco Lopera, Penelope K. Creagh, Laura B. Piñeros, Debjani Das, Martha L. Cervantes-Henríquez, Johan E. Acosta-López, Mario A. Isaza-Ruget, Lady G. Espinosa, Simon Easteal, Gustavo A. Quintero, Claudia Tamar Silva, Claudio A. Mastronardi, Mauricio Arcos-Burgos

**Affiliations:** 1 Genomics and Predictive Medicine Group, Department of Genome Sciences, John Curtin School of Medical Research, The Australian National University, Canberra, ACT 2600 Australia; 2 Universidad del Norte, Barranquilla, Colombia; 3 Neuroscience Research Group, University of Antioquia, Medellín, Colombia; 4 GENIUROS, Center for Research in Genetics and Genomics, Institute of Translational Medicine, School of Medicine and Health Sciences, Universidad del Rosario, Bogotá, Colombia; 5 Genome Diversity and Health Group, Department of Genome Sciences, John Curtin School of Medical Research, The Australian National University, ACT, Canberra, 2600 Australia; 6 Grupo de Neurociencias del Caribe, Universidad Simón Bolívar, Barranquilla, Colombia; 7 INPAC Research Group, Fundación Universitaria Sanitas, Bogotá, Colombia; 8 Studies in Translational Microbiology and Emerging Diseases (MICROS) Research Group, School of Medicine and Health Sciences, Universidad del Rosario, Bogotá, Colombia; 9 Neuroscience Group (NeUROS), Institute of Translational Medicine, School of Medicine and Health Sciences, Universidad del Rosario, Bogotá, Colombia

**Keywords:** Alzheimer’s disease, APOE*E2, Age of onset, *ASTN2*, Genetic isolate, *PSEN1*, Extreme phenotypes, *SNTG1*

## Abstract

The identification of novel genetic variants contributing to the widespread in the age of onset (AOO) of Alzheimer’s disease (AD) could aid in the prognosis and/or development of new therapeutic strategies focused on early interventions. We recruited 78 individuals with AD from the Paisa genetic isolate in Antioquia, Colombia. These individuals belong to the world largest multigenerational and extended pedigree segregating AD as a consequence of a dominant fully penetrant mutation in the *PSEN1* gene and exhibit an AOO ranging from the early 1930s to the late 1970s. To shed light on the genetic underpinning that could explain the large spread of the age of onset (AOO) of AD, 64 single nucleotide polymorphisms (SNP) associated with neuroanatomical, cardiovascular, and cognitive measures in AD were genotyped. Standard quality control and filtering procedures were applied, and single- and multi-locus linear mixed-effects models were used to identify AOO-associated SNPs. A full two-locus interaction model was fitted to define how identified SNPs interact to modulate AOO. We identified two key epistatic interactions between the APOE*E2 allele and SNPs *ASTN2*-rs7852878 and *SNTG1*-rs16914781 that delay AOO by up to ~ 8 years (95% CI 3.2–12.7, *P* = 1.83 × 10^−3^) and ~ 7.6 years (95% CI 3.3–11.8, *P* = 8.69 × 10^−4^), respectively, and validated our previous finding indicating that APOE*E2 delays AOO of AD in *PSEN1* E280 mutation carriers. This new evidence involving APOE*E2 as an AOO delayer could be used for developing precision medicine approaches and predictive genomics models to potentially determine AOO in individuals genetically predisposed to AD.

**Electronic supplementary material:**

The online version of this article (10.1007/s12035-018-1298-z) contains supplementary material, which is available to authorized users.

## Introduction

The prevalence of Alzheimer’s disease (AD) continues growing at an alarming pace. In 2006, the number of patients with AD was reported to be over 26.6 million worldwide, and it could rise by approximately fourfold to over 106.2 million by 2050 [1]. This neurodegenerative condition is incurable and constitutes a massive burden for patients, their families, and the public health system.

Genetic isolates have shown to be a powerful tool for the genetic mapping of inherited diseases [2]. For more than three decades, we have been studying the world’s largest known pedigree segregating AD in which the E280A (p.Glu280Ala) mutation in the *Presenilin-1* (*PSEN1*) gene causes early-onset AD [3, 4]. This pedigree is genetically homogeneous, exhibits a high degree of endogamy, and originated centuries ago as a consequence of a founder effect during the colonizing of Colombia by Spaniards [2–6]. To date, more than 5000 individuals descend from the original founder, 1784 have been enrolled in a comprehensive ongoing clinical monitoring study, and 1181 individuals have been genotyped (459 carry the *PSEN1* E280A mutation) [3]. Although the median Alzheimer’s disease age of onset (ADAOO) in this mentioned pedigree is ~ 49 years [3], it varies from the early 30s to the late 70s in some individuals [3, 7–10]. It is hypothesized that this substantial variation in the ADAOO is the result of interactions between *PSEN1* and other key genes to modify ADAOO, and that this modification results in some members of this pedigree developing signs and symptoms of AD at an earlier or later age than other members (that is, these gene interactions with *PSEN1* either accelerate or decelerate ADAOO).

In a recent study, we performed a pooling/resampling-based genome-wide association study (GWAS) and successfully identified both known and novel loci associated with ADAOO in individuals with the E280A mutation, including *DAOA*, *NPHP1*, *CLUAP1*, *EXOC2*, *CADPS2*, *GREM2*, and *CD44* [7]. Subsequent genetic studies in *PSEN1* E280A mutation carriers identified functional exonic variants within some of these genes [9] and demonstrated that the APOE*E2 allele (rs7412, *P* = 5.44 × 10^−35^, *P*_FDR_ = 2.13 × 10^−30^) delays ADAOO by ~ 12 years [8]. Interestingly, in a separate study, we also reported an exonic missense mutation in the *DAOA* gene (rs2391191, *P* = 1.94 × 10^−4^) that was found to delay the ADAOO in patients from the Paisa cohort in ~ 4 years [9]. It is also noteworthy to remark that the variant *SH3RF3-*rs6542814, flanking *NPHP1*, delays ADAOO by ~ 9 years [11], and the presence of two copies of the rare allele in *NPHP1-*rs906815 (rs906815, *P* = 4.51 × 10^−6^) accelerates ADAOO by ~ 21 years compared to the common allele in Caribbean Hispanic families carrying the *PSEN1* G206A mutation [12].

Since cognitive function and decline are highly polygenic traits where a large number of genetic factors of small effect are involved, it is difficult to find associations between these factors and clinical outcomes assessing cognition or cognitive decline [13, 14]. One of the standard methods to overcome this issue is to increase the sample size and subsequently increase the power to detect small effect sizes. Another possible approach is to perform targeted analysis by employing specific genetic markers that could be relevant to AD.

In the present study, we screened 78 individuals from the above-described pedigree and genotyped 65 single nucleotide polymorphisms (SNPs) previously reported to be associated with dementia and cognition. These SNPs showed association with neuroanatomical differences in brain areas that play essential roles in cognition such as the hippocampus, or that were related with hypertension because common genetic links appear to occur between AD and cardiovascular disease (Supplementary Table 1). We successfully replicated the association between the APOE*E2 allele and ADAOO, found two novel variants that also delay the age of onset of this pathological condition, and identified epistatic interactions between the APOE*E2 allele and variants within the *Astrotactin 2* (*ASTN2*) and *Syntrophin*, *Gamma 1* (*SNTG1*) genes that dramatically delay the ADAOO in *PSEN1* E280A mutation carriers.

## Methods

### Subjects

Seventy-eight individuals with AD (47 [60%] women, 31 men [40%]) carrying the *PSEN1* E280A mutation from the Metropolitan Area of Medellin in Antioquia, Colombia, were included in this study. Genetic studies have shown that this community has not been subject to microdifferentiation [2, 5]. Clinical, neurological, and neuropsychological assessments at the Group of Neurosciences AD Clinic used a Spanish version of The Consortium to Establish a Registry for Alzheimer’s Disease (CERAD) evaluation battery [15] adapted for the cultural and linguistic characteristics specific to this population [3, 16–18]. Mild cognitive impairment (MCI) and AD affection status were defined based on Petersen’s and DSM-IV criteria, respectively [19, 20]. The Ethics Committee of the University of Antioquia approved this study (Protocol 1115-408-20543). Informed consent was obtained from all participants.

### DNA Extraction and SNP Genotyping

Genomic DNA was extracted from peripheral blood, and whole-genome amplified, fragmented, hybridized, fluorescently tagged, and scanned using the Infinium assay [21]. Sixty-four SNPs were selected based on previous associations with dementia, cognition, neuroanatomical differences, and blood pressure (Supplementary Table 1), and further selected in our sample. Genomic DNA was normalized to a concentration of ~ 50 ng/μl, and 2.5 μL of genomic DNA was mixed with 2.5 μL TaqMan OpenArray Master Mix. The resulting samples were dispensed using the OpenArray® AccuFill™ System onto OpenArray plates with each plate containing 48 samples and 65 SNP assays per sample. The QuantStudio™ 12K Flex instrument (Applied Biosystems, Carlsbad, CA, USA) was used to perform the real-time PCR reactions on the loaded OpenArray plates. The fluorescence emission results were read using the OpenArray® SNP Genotyping Analysis software v1 (Applied Biosystems), and the genotyping analysis performed using TaqMan® Genotyper v1.3 with the auto call feature and the default settings.

### Genetic Association Analysis

Genotypes for the selected SNPs were processed, subject to quality control and association analysis performed using Golden Helix® SNP Variation Suite (SVS) 8.3.2 (Golden Helix, Inc. Bozeman, MT, USA). Quality control exclusion criteria included (i) deviations from Hardy-Weinberg equilibrium with *P* < 0.05/*m* (where *m* is the number of markers included for analysis), (ii) a minimum genotype call rate of 90%, (iii) the presence of one or more than two alleles, and (iv) a minor allele frequency (MAF) < 1% to exclude rare variants [22]. Genotype and allelic frequencies were estimated by maximum likelihood, and the identity by descent (IBD) matrix between all pairs of individuals was used for quality control.

Single- and multi-locus additive, dominant and recessive linear mixed-effect models (LMEMs) with up to 10 steps in the backward/forward optimization algorithm [23–25] were used to study the association between ADAOO and the aforementioned SNPs. The advantage of these models is the inclusion of both fixed (sex and years of education) and random effects, the latter to account for potential inbreeding (which, in our case, was estimated using the IBD matrix described above). A single-locus LMEM assumes that all loci have a small effect on the trait, while a multi-locus LMEM assumes that several loci have a large effect on the trait [25]. The optimal model was selected using a comprehensive exploration of multiple criteria (see [8–10] for more information). After the estimation procedure completed, the *P* values associated with the LMEM coefficients ${\widehat{\beta}}_1,{\widehat{\beta}}_2,\dots, {\widehat{\beta}}_{\mathrm{m}}$ were extracted and corrected for multiple testing using the false discovery rate (FDR) [26] and a method based on extreme-values theory [27].

### Effect of SNP × SNP Interactions on ADAOO

We evaluated potential SNP × SNP interactions between markers modifying ADAOO in carriers of the E280A mutation using a modified version of the full two-locus epistatic model [28–30]. Conceptually, the analysis of SNP **×** SNP interactions intends to determine whether the joint effect of two SNPs on the ADAOO is greater than that of either marker alone. For each pair of markers found to modify ADAOO in our patients, the ADAOO was compared at each genotype combination after correcting for potential confounding variables. Since the maximum number of genotype combinations is nine, it is likely that the sample size at each of these combinations is small. To overcome this, a nonparametric bootstrap [31, 32] procedure with *B* = 10,000 replicates was implemented to derive permutation-based *P* values for these comparisons.

## Results

### ADAOO Distribution

**Fig. 1:**
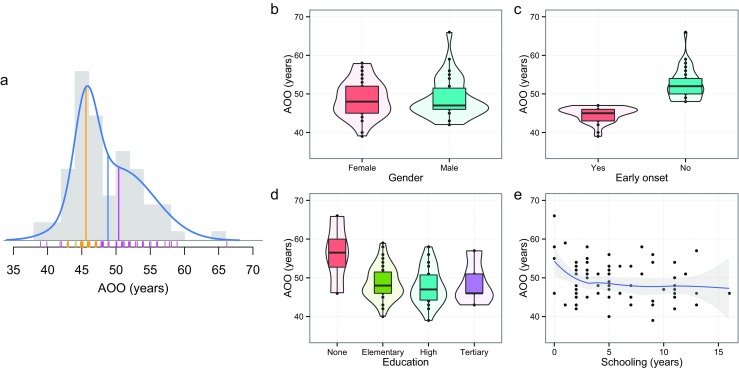
**a** ADAOO distribution in 78 patients with Alzheimer’s disease carrying the *PSEN1* E280A mutation. Notice the presence of two hidden groups with an average ADAOO of ~ 46 and ~ 51 years old, respectively. To identify these groups, a mixture of two Gaussian distributions was fitted as implemented in the mixtools [33] package for R [34]; the number of hidden groups was determined based on the log-likelihood criterion (the lowest the better). The blue vertical line is at ~ 48 years, which corresponds to the average ADAOO in our sample. Box and violin plots for the ADAOO by **b** gender, **c** early onset, and **d** education group. Only differences in the average ADAOO were found by AD status. **e** ADAOO as a function of the years of education. *AD* Alzheimer’s disease, *ADAOO* Alzheimer’s disease age of onset

The average ADAOO in all *PSEN1* E280A mutation carriers was 48.8 ± 4.9 years (blue vertical line, Fig. 1a). Mean ADAOO did not differ significantly by gender (*P* = 0.55, Fig. 1b). A total of 37 patients (20 women [54%] and 17 men [46%]) had an ADAOO < 48 years [7]. Years of education ranged between 0 and 16 years; four patients (5%) never attended school, 43 (55%) finished elementary school (grades 1 to 5), 26 (34%) finished high school (grades 6 to 11, inclusive), and 5 (6%) had tertiary education. The average ADAOO differed across education groups (*F*_3,74_ = 3.724, *P* = 0.015) (Fig. 1b). However, closer inspection of the data revealed that this effect was a consequence of the APOE*E2 allele in a 66-year-old male who never attended school. After excluding individuals that did not attend school, the effect of education groups on the ADAOO was no longer statistically significant (*F*_2,71_ = 0.373, *P* = 0.690). Thirty-seven (47%) individuals developed AD earlier than the average for this population (ADAOO < 48 years; early onset) and 41 developed late-onset AD (ADAOO ≥ 48 years). The average ADAOO was statistically different between these groups (early onset 44.8 ± 1.9, late onset 52.5 ± 3.9, *P* < 2.5 × 10^−16^, Fig. 1b). No association between gender (*P* = 0.979, Fig. 1b) or years of education was found (*R*^2^ = 0.028, *P* = 0.076, Fig. 1b).

### ADAOO-Associated SNPs

**Fig. 2:**
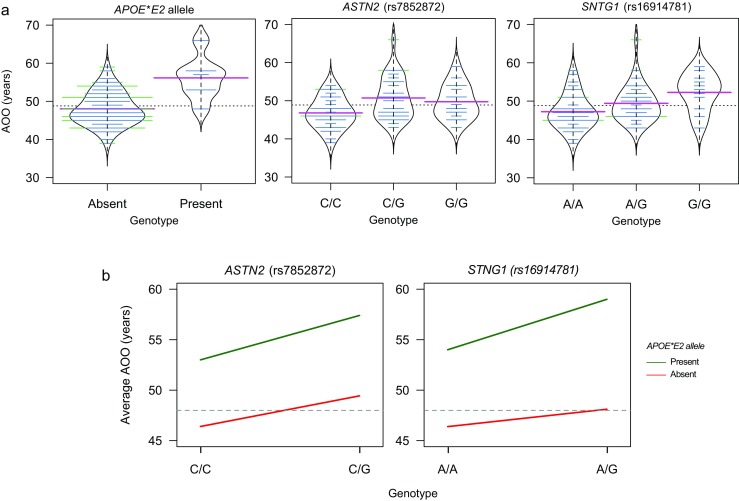
**a** Effect of the presence of the APOE*E2 allele, and the genotypes in rs7852872-*ASTN2* and rs16914781-*SNTG1* on ADAOO. A two-sample *t* test indicates the presence of the APOE*E2 allele increases the ADAOO by ~ 8.1 years (*t*_72_ = 4.67, 95% CI 4.6–11.6, *P* = 1.37 × 10^−6^). Pink, blue, and dotted horizontal lines are, respectively, the within genotype average ADAOO, the individuals’ ADAOO, and the global average ADAOO in our sample. **b** Effect of the APOE*E2×*ASTN2* and APOE*E2×*SNTG1* interactions on ADAOO. Green lines symbolize protection, red lines susceptibility, and the gray line the average ADAOO in our sample. Note that the APOE*E2 allele delays ADAOO regardless of the interacting marker. Abbreviations as in Fig. 1

A dominant multi-locus LMEM with three steps in the forward/backward selection algorithm [25] was selected based on the *mPPA* and pseudo-heritability criteria. This oligogenic model includes variants rs7412 (*APOE*, *P* = 1.94 × 10^−4^, *P*_FDR_ = 9.34 × 10^−3^, Table 1), rs7852878 (*ASTN2*, *P* = 1.94 × 10^−4^, *P*_FDR_ = 9.34 × 10^−3^, Table 1), and rs16914781 (*SNTG1*, *P* = 1.94 × 10^−4^, *P*_FDR_ = 9.34 × 10^−3^, Table 1), which explains ~ 43% of the ADAOO variance. The proportion of the ADAOO variance explained by each marker is ~ 24, ~ 13, and ~ 8% for rs7412, rs7852872, and rs16914781, respectively. No gender- or education-specific effect of these SNPs was found (Table 1). Because all estimated *β* coefficients from this model are positive (Table 1), these alleles delay the ADAOO in our sample of *PSEN1* E280A mutation carriers. In particular, individuals with the C/T genotype in *APOE*-rs7412 (that is, the APOE*E2 allele) have an ADAOO ~ 8 years later than that of individuals with the C/C genotype ($\widehat{\beta}=8.21,{\widehat{\mathrm{SE}}}_{\widehat{\beta}}$= 1.5; Table 1a and Fig. 2a). Likewise, *PSEN1* E280A mutation carriers with C/G or G/G in *ASTN2*-rs7852878 have an ADAOO ~ 3.7 years later compared to that of C/C individuals ($\widehat{\beta} = 3.68,{\widehat{\mathrm{SE}}}_{\widehat{\beta}}$= 0.88; Table 1 and Fig. 2a). In addition, members of this pedigree with the G/G genotype in *SNTG1*-rs16914781 have a ~ 3.3 years delay in the ADAOO compared to those with A/A or A/G ($\widehat{\beta}=3.27,{\widehat{\mathrm{SE}}}_{\widehat{\beta}}$= 0.872; Table 1 and Fig. 2a).

**Table 1:** Results of the association analysis for ADAOO in 78 patients with *PSEN1* E280A Alzheimer’s disease (a). Proportion of variance explained and gender- and education-specific effects of ADAOO associated SNPs (b)

(a)										
Chr	SNP^a^	Position	Gene	Marker information				Multi-locus linear mixed-effects model		
				Ref/Alt	MA (Freq)	CR	Change	*β* (SE_*β*_)	*P*	*P*_FDR_
19	rs7412	45,412,078	*APOE*	C/T	T (0.046)	0.974	p.Arg176Cys	8.213 (1.505)	6.48 × 10^−7^	4.21 × 10^−5^
9	rs7852872	119,249,338	*ASTN2*	C/G	G (0.396)	0.987	Intronic	3.684 (0.881)	8.10 × 10^−5^	2.63 × 10^−3^
8	rs16914781	51,287,481	*SNTG1*	A/G	G (0.339)	1.000	Intronic	3.273 (0.872)	3.52 × 10^−4^	7.62 × 10^−3^
(b)										
SNP^a^	PVE	Sex			Education group					
		*χ* ^2^	*df*	*P*	*χ* ^2^	*df*	*P*			
rs7412	0.239	0.023	2	0.989	4.303	6	0.636			
rs7852872	0.133	1.041	1	0.308	2.681	3	0.443			
rs16914781	0.076	0.939	2	0.625	6.331	6	0.387			

^a^UCSC GRCh37/hg19 coordinates

*ADAOO* Alzheimer’s disease age of onset, *Chr* chromosome, *SNP* single nucleotide polymorphism, *Ref/Alt* reference/alternate allele, *MA* minor allele, *Freq* frequency, *CR* call rate, *β* regression coefficient, *SE*_*β*_ standard error of *β*, *P P* value, *FDR* false discovery rate, *PVE* proportion of variance explained, *χ*^2^ test statistic, *df* degrees of freedom

### Effect of the APOE*E2×*ASTN2* and APOE*E2×*SNTG1* Interactions on ADAOO

The presence of the APOE*E2 allele in E280A mutation carriers was found to delay ADAOO by ~ 8.1 years (95% CI 4.65–11.58, *P* = 1.37 × 10^−5^) (Fig. 2b). A similar effect was observed when this same allele interacts with markers *ASTN2*-rs7852878 and *SNTG1*-rs16914781, which suggests an epistatic mechanism between APOE*E2 and *ASTN2* (Fig. 2b), and between APOE*E2 and *SNTG1* (Fig. 2b) to modify the ADAOO in carriers of the E280A mutation. In particular, the ADAOO in individuals with the APOE*E2 allele and C/G genotype in *ASTN2*-rs7852878 is ~ 8 years (95% CI 3.2–12.7, *P* = 1.83 × 10^−3^) later than that of individuals lacking the APOE*E2 allele (Fig. 2b). Similarly, those with C/C in *ASTN2*-rs7852878 carrying the APOE*E2 allele have an ADAOO ~ 6.6 years (9% CI 1.2–11.9, *P* = 0.017) later compared to non-carriers (Fig. 2b). Conversely, individuals with the A/A genotype in *SNTG1*-rs16914781 carrying the APOE*E2 allele have an ADAOO ~ 7.6 years (95% CI 3.3–11.8, *P* = 8.69 × 10^−4^) later than that observed in non-carriers (Fig. 2b), and the presence of the APOE*E2 allele delayed the ADAOO in ~ 11 years (95% CI 6.6–15.2, *P* = 1.7 × 10^−5^) in individuals with the A/G genotype in *SNTG1*-rs16914781 (Fig. 2b). We found no effect of the *ASTN2*×*SNTG1* interaction on the ADAOO.

## Discussion

In this study, we targeted neuroanatomical, cardiovascular, and cognitive-associated markers in familial AD from the Paisa community, a genetic isolate from Antioquia, Colombia. Even though several GWAS studies have provided a potential list of a handful of putative candidate genes for sporadic AD (i.e., an age of onset > 65 years), most of those genes failed in their replication. It is well known that heterogeneity of genetic and environmental background could largely account for this apparent discrepancy. Thus, to increase power in our analyses, our approach was aimed at performing a targeted analysis in a multigenerational family from a local community that is exposed to a quite homogenous environment. More specifically, we employed 65 genetic markers related to Alzheimer’s disease in a large family from the local Paisa community that originated from a common ancestor from Northern Spain during the 1500s. In this community, Alzheimer’s disease is quite common as a result of the high frequency of the autosomal dominant and fully penetrant *PSEN1* E280A allele. Our main goal was to shed light on the genetic underpinning that could explain the large spread of the age of onset of AD that ranks from the early 1930s to late 1970s.

This cohort was also subjected to two earlier preliminary studies in which smaller sample sizes were employed, and different outcomes were observed [35, 36]. Since the time those studies were performed, more E280A carriers have been identified. Hence, here we expanded the sample to detect new genes that could explain the widespread of the ADAOO observed in E280A carriers. Our present data show that the presence of the APOE*E2 allele confers protection by delaying the ADAOO by ~ 8.2 years (95% CI 5.2–11.2, *P* = 4.21 × 10^−5^; Fig. 2a), which confirms our most recent reported finding in a sample of 71 *PSEN1* E280A mutation carriers displaying an extreme ADAOO [8]. Basically, by increasing the sample size to 78 patients carrying the E280A mutation, in the present study, we corroborated the decelerating APOE*E2 effect on ADAOO previously shown in individuals from the Paisa community [8]. Power analyses indicate that, overall, the ADAOO can be safely tested using our current sample size (see Supplementary Material).

Collectively, previous and current work in this genetic isolate suggests that the ADAOO accelerating and decelerating effects conferred by the APOE*E4 and APOE*E2 alleles, respectively, become evident. Therefore, our results provide convincing evidence that not only does the APOE*E2 allele exert a protective role in the onset of AD in sporadic patients [37, 38], but also in the *PSEN1* E280A familial cases.

The role of beta-amyloid (Aβ) in AD has been openly challenged [39–41]. One of the primary reasons is that there is evidence showing that Aβ deposition rises with healthy aging and its increase is not necessarily correlated with the onset of dementia and the progression to AD [41, 42]. However, it is noteworthy to remark that patients with familial Alzheimer’s disease display fibrillar Aβ pathology several years before symptoms onset [43]. For instance, by employing florbetapir PET analyses, Fleisher et al. showed that individuals from the Antioquia cohort carrying the *PSEN1* E280A mutation showed evident accumulation of fibrillar Aβ at a mean age of 28.2 years, which was approximately 16 and 21 years before the expected MCI and dementia onset, respectively [44]. Thus, it appears that fibrillar Aβ pathology could represent an early preclinical stage of AD. Another piece of evidence supporting that Aβ is involved in the pathogenesis of AD is the fact that the three well-known genes that cause a dominant Mendelian form of familial AD (*APP*, *PSEN1*, and *PSEN2*) are involved in the processing of Aβ peptides [45–47]. Aβ peptides vary between 37 and 43 amino acids in length depending on the γ-secretase cleavage site. Mounting evidence suggests that the majority of early-onset familial AD mutations in *APP*, *PSEN1*, and *PSEN2* elevate the Aβ1–42:Aβ1–40 ratio, which favors the aggregation of neurotoxic oligomeric assemblies of Aβ. It is considered that Aβ1–42 is more amyloidogenic than other Aβ peptides, which assemble into soluble Aβ oligomers that are thought to cause synaptic loss and a progressive cognitive decline in AD [48]. Aβ1–42 oligomers can elicit an inflammatory cascade by triggering the activation of microglia [49]. Moreover, Aβ oligomers associate with membrane proteins in synapses [50] and astrocytes [51]. In post-synaptic, neurons increase the Ca^2+^ concentration causing inflammation and cell death [50]. Post-mortem studies carried in brain tissue from the E280A kindred suggest that their *PSEN1* mutation selectively increases the processing of the amyloidogenic peptide Aβ1–42 [47]. Mounting evidence suggests that there are links between Aβ and tau in the pathogenesis of AD [52–54]. Aβ promotes abnormal tau phosphorylation and aggregation into neurofibrillary tangles, which is associated with neuronal toxicity and impaired cognition in AD. For instance, in functional studies employing transgenic animal models and neuronal cell culture, it was found that a 56-kDa amyloid oligomer elicited an influx in intracellular Ca2+ that triggered phosphorylation of tau at a site that promoted its aggregation [55]. This recent finding expands previous evidence supporting a possible link between Aβ and tau in the pathogenesis of AD [52–54].

In this context, it can be argued that the APOE*E2 variant might cause a beneficial impact on AD by improving the clearance of central Aβ, and consequently delay the onset of AD [56]. On the other hand, the APOE*E4 variant accelerates the ADAOO since it performs poorly in the clearance of Aβ peptides thereby favoring the formation of aggregates and the occurrence of the disease [57, 58].

Marker rs7852878, harbored in *ASTN2*, was also found to delay ADAOO in individuals with AD carrying the E280A mutation. ASTN2 is an integral membrane protein that participates in glial-guided neuronal migrations and is largely expressed within the hippocampus [59]. Genomic variants in genes engaged in neuronal migration processes have been linked to several neurocognitive and psychiatric disorders. For instance, genes casually linked to schizophrenia such as *Disrupted in schizophrenia-1* (*DISC1*), *Reelin, neuroregulin* (*NRG*), and its receptor, *ERBB4*, control neuronal migration during brain development [60]. Likewise, genes linked to ADHD (*LPHN3*) [61, 62], autism (*YWHAZ*) [63], and depressive behavior (*BDNF*) [64] also control neuronal fate within different brain regions. Interestingly, SNPs within *ASTN2* have been associated with cognitive decline and reduced hippocampal volume [65, 66] and several psychiatric conditions such as schizophrenia [67, 68], ADHD [69], and bipolar disorder [68]. More recently, genetic variants within *ASTN2* have been associated with ADAOO in late-onset AD [70].

We found that marker rs16914781 within *SNTG1* delays ADAOO by ~ 3.2 years in individuals carrying the *PSEN1* E280A mutation (Table 1). SNTG1 belongs to the syntrophin family; it is an adapter protein that participates in the subcellular organization of several proteins. It also mediates gamma-enolase trafficking to the plasma membrane and is involved in neurotrophic signaling [71]. SNTG1 is expressed exclusively in neurons, including Purkinje cells, hippocampal pyramidal cells, and in multiple cortical regions, where it could be playing important roles in the pathophysiology of AD and other neurodegenerative/neuropsychiatric conditions [59, 72]. *SNTG1* has been reported as a highly penetrant recessive locus in schizophrenia [72], and as AOO modifier gene in AD [7]. More recently, a circular RNA hotspot involving *SNTG1* has recently been identified in multiple system atrophy (MSA) [73], a neurodegenerative disorder causing parkinsonism, cerebellar ataxia, and autonomic, urogenital, and pyramidal dysfunction in various combinations. Previously, a case report displayed an association of MSA and AD [74]. *SNTG1* has also been implicated in obstructive sleep apnea [75], a condition that is highly prevalent in patients with Alzheimer’s disease [76].

To the best of our knowledge, we are the first to demonstrate a significant association between variants within *ASTN2* and *SNTG1*, and ADAOO in individuals with familial AD caused by a fully penetrant mutation. Our study suggests that the genetic variants described here exert a protective effect by delaying ADAOO up to ~ 3.7 years (Table 1); this value increases to ~ 11 years when the APOE*E2 allele is present (Fig. 2a). Future studies need to be performed to address the underlying action mechanism describing the interaction between *ASTN2* and *PSEN1*, and between *STNG1* and *PSEN1*.

## Electronic supplementary material

ESM 1 (DOCX 331 kb)

## References

[1] Brookmeyer R, Johnson E, Ziegler-Graham K, Arrighi HM (2007). Forecasting the global burden of Alzheimer’s disease. Alzheimers Dement.

[2] Arcos-Burgos M, Muenke M (2002). Genetics of population isolates. Clin Genet.

[3] Acosta-Baena N, Sepulveda-Falla D, Lopera-Gomez CM, Jaramillo-Elorza MC, Moreno S, Aguirre-Acevedo DC (2011). Pre-dementia clinical stages in presenilin 1 E280A familial early-onset Alzheimer’s disease: a retrospective cohort study. Lancet Neurol.

[4] Lopera F, Ardilla A, Martinez A, Madrigal L, Arango-Viana JC, Lemere CA (1997). Clinical features of early-onset Alzheimer disease in a large kindred with an E280A presenilin-1 mutation. JAMA.

[5] Bravo ML, Valenzuela CY, Arcos-Burgos OM (1996). Polymorphisms and phyletic relationships of the Paisa community from Antioquia (Colombia). Gene Geogr.

[6] Londono AC, Castellanos FX, Arbelaez A, Ruiz A, Aguirre-Acevedo DC, Richardson AM, Easteal S, Lidbury BA, Arcos-Burgos M, Lopera F (2014). An 1H-MRS framework predicts the onset of Alzheimer’s disease symptoms in PSEN1 mutation carriers. Alzheimers Dement.

[7] Velez JI, Chandrasekharappa SC, Henao E, Martinez AF, Harper U, Jones M (2013). Pooling/bootstrap-based GWAS (pbGWAS) identifies new loci modifying the age of onset in PSEN1 p.Glu280Ala Alzheimer’s disease. Mol Psychiatry.

[8] Velez JI, Lopera F, Sepulveda-Falla D, Patel HR, Johar AS, Chuah A (2016). APOE*E2 allele delays age of onset in PSEN1 E280A Alzheimer’s disease. Mol Psychiatry.

[9] Velez JI, Rivera D, Mastronardi CA, Patel HR, Tobon C, Villegas A (2016). A mutation in DAOA modifies the age of onset in PSEN1 E280A Alzheimer’s disease. Neural Plast.

[10] Velez JI, Lopera F, Patel HR, Johar AS, Cai Y, Rivera D (2016). Mutations modifying sporadic Alzheimer’s disease age of onset. Am J Med Genet B Neuropsychiatr Genet.

[11] Lee JH, Cheng R, Vardarajan BN, Lantigua RA, Reyes-Dumeyer D, Ortmann W, Graham R, Bhangale T, Behrens T, Medrano M, Jimenez-Velázquez I, Mayeux R (2014). SORBS2, SH3RF3, and NPHP1 modify age at onset in carriers of the G206A mutation in PSEN1 with familial Alzheimer’s disease. Alzheimers Dement.

[12] Lee JH, Cheng R, Vardarajan B, Lantigua R, Reyes-Dumeyer D, Ortmann W, Graham RR, Bhangale T, Behrens TW, Medrano M, Jiménez-Velázquez IZ, Mayeux R (2015). Genetic modifiers of age at onset in carriers of the G206A mutation in PSEN1 with familial Alzheimer disease among Caribbean Hispanics. JAMA Neurol.

[13] Chabris CF, Hebert BM, Benjamin DJ, Beauchamp J, Cesarini D, van der Loos M, Johannesson M, Magnusson PKE, Lichtenstein P, Atwood CS, Freese J, Hauser TS, Hauser RM, Christakis N, Laibson D (2012). Most reported genetic associations with general intelligence are probably false positives. Psychol Sci.

[14] Davies G, Tenesa A, Payton A, Yang J, Harris SE, Liewald D, Ke X, le Hellard S, Christoforou A, Luciano M, McGhee K, Lopez L, Gow AJ, Corley J, Redmond P, Fox HC, Haggarty P, Whalley LJ, McNeill G, Goddard ME, Espeseth T, Lundervold AJ, Reinvang I, Pickles A, Steen VM, Ollier W, Porteous DJ, Horan M, Starr JM, Pendleton N, Visscher PM, Deary IJ (2011). Genome-wide association studies establish that human intelligence is highly heritable and polygenic. Mol Psychiatry.

[15] Morris JC, Heyman A, Mohs RC, Hughes JP, van Belle G, Fillenbaum G, Mellits ED, Clark C (1989). The consortium to establish a registry for Alzheimer's disease (CERAD). Part I. Clinical and neuropsychological assessment of Alzheimer’s disease. Neurology.

[16] Fleisher AS, Chen K, Quiroz YT, Jakimovich LJ, Gomez MG, Langois CM, Langbaum JBS, Ayutyanont N, Roontiva A, Thiyyagura P, Lee W, Mo H, Lopez L, Moreno S, Acosta-Baena N, Giraldo M, Garcia G, Reiman RA, Huentelman MJ, Kosik KS, Tariot PN, Lopera F, Reiman EM (2012). Florbetapir PET analysis of amyloid-beta deposition in the presenilin 1 E280A autosomal dominant Alzheimer's disease kindred: a cross-sectional study. Lancet Neurol.

[17] Reiman EM, Quiroz YT, Fleisher AS, Chen K, Velez-Pardo C, Jimenez-Del-Rio M (2012). Brain imaging and fluid biomarker analysis in young adults at genetic risk for autosomal dominant Alzheimer’s disease in the presenilin 1 E280A kindred: a case-control study. Lancet Neurol.

[18] Reiman EM, Langbaum JB, Fleisher AS, Caselli RJ, Chen K, Ayutyanont N (2011). Alzheimer’s prevention initiative: a plan to accelerate the evaluation of presymptomatic treatments. J Alzheimers Dis.

[19] Petersen RC, Smith GE, Waring SC, Ivnik RJ, Tangalos EG, Kokmen E (1999). Mild cognitive impairment: clinical characterization and outcome. Arch Neurol.

[20] Association AP. American Psychiatric Association: diagnostic and statistical manual of mental disorders. Fourth ed. Association AP, editor. Washington, D.C.2000.

[21] Gunderson KL, Steemers FJ, Lee G, Mendoza LG, Chee MS (2005). A genome-wide scalable SNP genotyping assay using microarray technology. Nat Genet.

[22] Bansal V, Libiger O, Torkamani A, Schork NJ (2010). Statistical analysis strategies for association studies involving rare variants. Nat Rev Genet.

[23] Liu DJ, Leal SM (2010). A novel adaptive method for the analysis of next-generation sequencing data to detect complex trait associations with rare variants due to gene main effects and interactions. PLoS Genet.

[24] Liu DJ, Leal SM (2010). Replication strategies for rare variant complex trait association studies via next-generation sequencing. Am J Hum Genet.

[25] Segura V, Vilhjalmsson BJ, Platt A, Korte A, Seren U, Long Q (2012). An efficient multi-locus mixed-model approach for genome-wide association studies in structured populations. Nat Genet.

[26] Benjamini Y, Hochberg Y (1995). Controlling the false discovery rate: a practical and powerful approach to multiple testing. J R Stat Soc Ser B Methodol.

[27] Vélez JI, Correa JC, Arcos-Burgos M (2014). A new method for detecting significant p-values with applications to genetic data. Revista Colombiana de Estadistica.

[28] Acosta MT, Velez JI, Bustamante ML, Balog JZ, Arcos-Burgos M, Muenke M (2011). A two-locus genetic interaction between LPHN3 and 11q predicts ADHD severity and long-term outcome. Transl Psychiatry.

[29] Cordell HJ (2002). Epistasis: what it means, what it doesn’t mean, and statistical methods to detect it in humans. Hum Mol Genet.

[30] Cordell HJ, Todd JA, Hill NJ, Lord CJ, Lyons PA, Peterson LB, Wicker LS, Clayton DG (2001). Statistical modeling of interlocus interactions in a complex disease: rejection of the multiplicative model of epistasis in type 1 diabetes. Genetics.

[31] Efron B (1979). Bootstrap methods: another look at the Jacknife. Ann Stat.

[32] Efron B, Tibshirani R (1986). Bootstrap methods for standard errors, confidence intervals and other measures of statistical accuracy. Stat Sci.

[33] Benaglia T, Chauveau D, Hunter DR, Young D (2009). Mixtools: an R package for analyzing finite mixture models. J Stat Softw.

[34] R Core Team. R: a language and environment for statistical computing. Vienna, Austria: R Foundation for Statistical Computing; 2018.

[35] Lendon CL, Martinez A, Behrens IM, Kosik KS, Madrigal L, Norton J, Neuman R, Myers A, Busfield F, Wragg M, Arcos M, Viana JCA, Ossa J, Ruiz A, Goate AM, Lopera F (1997). E280A PS-1 mutation causes Alzheimer’s disease but age of onset is not modified by ApoE alleles. Hum Mutat.

[36] Pastor P, Roe CM, Villegas A, Bedoya G, Chakraverty S, Garcia G (2003). Apolipoprotein Eepsilon4 modifies Alzheimer’s disease onset in an E280A PS1 kindred. Ann Neurol.

[37] Corder EH, Saunders AM, Risch NJ, Strittmatter WJ, Schmechel DE, Gaskell PC (1994). Protective effect of apolipoprotein E type 2 allele for late onset Alzheimer disease. Nat Genet.

[38] Berlau DJ, Corrada MM, Head E, Kawas CH (2009). APOE epsilon2 is associated with intact cognition but increased Alzheimer pathology in the oldest old. Neurology.

[39] Verdile G, Fuller S, Atwood CS, Laws SM, Gandy SE, Martins RN (2004). The role of beta amyloid in Alzheimer’s disease: still a cause of everything or the only one who got caught?. Pharmacol Res.

[40] Brier Matthew R., Gordon Brian, Friedrichsen Karl, McCarthy John, Stern Ari, Christensen Jon, Owen Christopher, Aldea Patricia, Su Yi, Hassenstab Jason, Cairns Nigel J., Holtzman David M., Fagan Anne M., Morris John C., Benzinger Tammie L. S., Ances Beau M. (2016). Tau and Aβ imaging, CSF measures, and cognition in Alzheimer’s disease. Science Translational Medicine.

[41] Lee HG, Casadesus G, Zhu X, Takeda A, Perry G, Smith MA (2004). Challenging the amyloid cascade hypothesis: senile plaques and amyloid-beta as protective adaptations to Alzheimer disease. Ann N Y Acad Sci.

[42] Fjell AM (2014). McEvoy L, Holland D, Dale AM, Walhovd KB, Alzheimer’s disease neuroimaging I. What is normal in normal aging? Effects of aging, amyloid and Alzheimer’s disease on the cerebral cortex and the hippocampus. Prog Neurobiol.

[43] Bateman RJ, Xiong C, Benzinger TLS, Fagan AM, Goate A, Fox NC, Marcus DS, Cairns NJ, Xie X, Blazey TM, Holtzman DM, Santacruz A, Buckles V, Oliver A, Moulder K, Aisen PS, Ghetti B, Klunk WE, McDade E, Martins RN, Masters CL, Mayeux R, Ringman JM, Rossor MN, Schofield PR, Sperling RA, Salloway S, Morris JC, Dominantly Inherited Alzheimer Network (2012). Clinical and biomarker changes in dominantly inherited Alzheimer’s disease. N Engl J Med.

[44] Fleisher AS, Chen K, Quiroz YT, Jakimovich LJ, Gomez MG, Langois CM, Langbaum JBS, Ayutyanont N, Roontiva A, Thiyyagura P, Lee W, Mo H, Lopez L, Moreno S, Acosta-Baena N, Giraldo M, Garcia G, Reiman RA, Huentelman MJ, Kosik KS, Tariot PN, Lopera F, Reiman EM (2012). Florbetapir PET analysis of amyloid-beta deposition in the presenilin 1 E280A autosomal dominant Alzheimer’s disease kindred: a cross-sectional study. Lancet Neurol.

[45] Guerreiro RJ, Gustafson DR, Hardy J (2012). The genetic architecture of Alzheimer’s disease: beyond APP, PSENs and APOE. Neurobiol Aging.

[46] Sun X, Chen WD, Wang YD (2015). beta-amyloid: the key peptide in the pathogenesis of Alzheimer’s disease. Front Pharmacol.

[47] Lemere CA, Lopera F, Kosik KS, Lendon CL, Ossa J, Saido TC, Yamaguchi H, Ruiz A, Martinez A, Madrigal L, Hincapie L, Arango L. JC, Anthony DC, Koo EH, Goate AM, Selkoe DJ, Arango V. JC (1996). The E280A presenilin 1 Alzheimer mutation produces increased A beta 42 deposition and severe cerebellar pathology. Nat Med.

[48] Selkoe DJ (2008). Soluble oligomers of the amyloid beta-protein impair synaptic plasticity and behavior. Behav Brain Res.

[49] Crouse NR, Ajit D, Udan ML, Nichols MR (2009). Oligomeric amyloid-beta(1-42) induces THP-1 human monocyte adhesion and maturation. Brain Res.

[50] Dinamarca MC, Rios JA, Inestrosa NC (2012). Postsynaptic receptors for amyloid-beta oligomers as mediators of neuronal damage in Alzheimer’s disease. Front Physiol.

[51] Walker D, Lue LF, Paul G, Patel A, Sabbagh MN (2015). Receptor for advanced glycation endproduct modulators: a new therapeutic target in Alzheimer’s disease. Expert Opin Investig Drugs.

[52] Noble W, Hanger DP, Miller CC, Lovestone S (2013). The importance of tau phosphorylation for neurodegenerative diseases. Front Neurol.

[53] Zempel H, Thies E, Mandelkow E, Mandelkow EM (2010). Abeta oligomers cause localized Ca(2+) elevation, missorting of endogenous Tau into dendrites, Tau phosphorylation, and destruction of microtubules and spines. J Neurosci.

[54] Bloom GS (2014). Amyloid-beta and tau: the trigger and bullet in Alzheimer disease pathogenesis. JAMA Neurol.

[55] Amar F, Sherman MA, Rush T, Larson M, Boyle G, Chang L (2017). Amyloid-β oligomer Aβ*56 induces specific alterations of tau phosphorylation and neuronal signaling. Sci Signal.

[56] Suri S, Heise V, Trachtenberg AJ, Mackay CE (2013). The forgotten APOE allele: a review of the evidence and suggested mechanisms for the protective effect of APOE varepsilon2. Neurosci Biobehav Rev.

[57] Chalmers K, Wilcock GK, Love S (2003). APOE epsilon 4 influences the pathological phenotype of Alzheimer’s disease by favouring cerebrovascular over parenchymal accumulation of A beta protein. Neuropathol Appl Neurobiol.

[58] Kanekiyo T, Xu H, Bu G (2014). ApoE and Abeta in Alzheimer’s disease: accidental encounters or partners?. Neuron.

[59] Lukk M, Kapushesky M, Nikkila J, Parkinson H, Goncalves A, Huber W (2010). A global map of human gene expression. Nat Biotechnol.

[60] Kamiya A, Kubo K, Tomoda T, Takaki M, Youn R, Ozeki Y, Sawamura N, Park U, Kudo C, Okawa M, Ross CA, Hatten ME, Nakajima K, Sawa A (2005). A schizophrenia-associated mutation of DISC1 perturbs cerebral cortex development. Nat Cell Biol.

[61] Arcos-Burgos M, Jain M, Acosta MT, Shively S, Stanescu H, Wallis D, Domené S, Vélez JI, Karkera JD, Balog J, Berg K, Kleta R, Gahl WA, Roessler E, Long R, Lie J, Pineda D, Londoño AC, Palacio JD, Arbelaez A, Lopera F, Elia J, Hakonarson H, Johansson S, Knappskog PM, Haavik J, Ribases M, Cormand B, Bayes M, Casas M, Ramos-Quiroga JA, Hervas A, Maher BS, Faraone SV, Seitz C, Freitag CM, Palmason H, Meyer J, Romanos M, Walitza S, Hemminger U, Warnke A, Romanos J, Renner T, Jacob C, Lesch KP, Swanson J, Vortmeyer A, Bailey-Wilson JE, Castellanos FX, Muenke M (2010). A common variant of the latrophilin 3 gene, LPHN3, confers susceptibility to ADHD and predicts effectiveness of stimulant medication. Mol Psychiatry.

[62] O'Sullivan ML, de Wit J, Savas JN, Comoletti D, Otto-Hitt S, Yates JR (2012). FLRT proteins are endogenous latrophilin ligands and regulate excitatory synapse development. Neuron.

[63] Toma C, Torrico B, Hervas A, Valdes-Mas R, Tristan-Noguero A, Padillo V (2014). Exome sequencing in multiplex autism families suggests a major role for heterozygous truncating mutations. Mol Psychiatry.

[64] Lee BH, Kim YK (2010). The roles of BDNF in the pathophysiology of major depression and in antidepressant treatment. Psychiatry Investig.

[65] Bis JC, DeCarli C, Smith AV, van der Lijn F, Crivello F, Fornage M, Debette S, Shulman JM, Schmidt H, Srikanth V, Schuur M, Yu L, Choi SH, Sigurdsson S, Verhaaren BF, DeStefano A, Lambert JC, Jack CR Jr, Struchalin M, Stankovich J, Ibrahim-Verbaas CA, Fleischman D, Zijdenbos A, den Heijer T, Mazoyer B, Coker LH, Enzinger C, Danoy P, Amin N, Arfanakis K, van Buchem M, de Bruijn RF, Beiser A, Dufouil C, Huang J, Cavalieri M, Thomson R, Niessen WJ, Chibnik LB, Gislason GK, Hofman A, Pikula A, Amouyel P, Freeman KB, Phan TG, Oostra BA, Stein JL, Medland SE, Vasquez AA, Hibar DP, Wright MJ, Franke B, Martin NG, Thompson PM, Nalls MA, Uitterlinden AG, Au R, Elbaz A, Beare RJ, van Swieten J, Lopez OL, Harris TB, Chouraki V, Breteler MM, de Jager PL, Becker JT, Vernooij MW, Knopman D, Fazekas F, Wolf PA, van der Lugt A, Gudnason V, Longstreth WT Jr, Brown MA, Bennett DA, van Duijn C, Mosley TH, Schmidt R, Tzourio C, Launer LJ, Ikram MA, Seshadri S, Enhancing Neuro Imaging Genetics through Meta-Analysis Consortium, Cohorts for Heart and Aging Research in Genomic Epidemiology Consortium (2012). Common variants at 12q14 and 12q24 are associated with hippocampal volume. Nat Genet.

[66] Hibar DP, Adams HHH, Jahanshad N, Chauhan G, Stein JL, Hofer E, Renteria ME, Bis JC, Arias-Vasquez A, Ikram MK, Desrivières S, Vernooij MW, Abramovic L, Alhusaini S, Amin N, Andersson M, Arfanakis K, Aribisala BS, Armstrong NJ, Athanasiu L, Axelsson T, Beecham AH, Beiser A, Bernard M, Blanton SH, Bohlken MM, Boks MP, Bralten J, Brickman AM, Carmichael O, Chakravarty MM, Chen Q, Ching CRK, Chouraki V, Cuellar-Partida G, Crivello F, den Braber A, Doan NT, Ehrlich S, Giddaluru S, Goldman AL, Gottesman RF, Grimm O, Griswold ME, Guadalupe T, Gutman BA, Hass J, Haukvik UK, Hoehn D, Holmes AJ, Hoogman M, Janowitz D, Jia T, Jørgensen KN, Karbalai N, Kasperaviciute D, Kim S, Klein M, Kraemer B, Lee PH, Liewald DCM, Lopez LM, Luciano M, Macare C, Marquand AF, Matarin M, Mather KA, Mattheisen M, McKay DR, Milaneschi Y, Muñoz Maniega S, Nho K, Nugent AC, Nyquist P, Loohuis LMO, Oosterlaan J, Papmeyer M, Pirpamer L, Pütz B, Ramasamy A, Richards JS, Risacher SL, Roiz-Santiañez R, Rommelse N, Ropele S, Rose EJ, Royle NA, Rundek T, Sämann PG, Saremi A, Satizabal CL, Schmaal L, Schork AJ, Shen L, Shin J, Shumskaya E, Smith AV, Sprooten E, Strike LT, Teumer A, Tordesillas-Gutierrez D, Toro R, Trabzuni D, Trompet S, Vaidya D, van der Grond J, van der Lee SJ, van der Meer D, van Donkelaar MMJ, van Eijk KR, van Erp TGM, van Rooij D, Walton E, Westlye LT, Whelan CD, Windham BG, Winkler AM, Wittfeld K, Woldehawariat G, Wolf C, Wolfers T, Yanek LR, Yang J, Zijdenbos A, Zwiers MP, Agartz I, Almasy L, Ames D, Amouyel P, Andreassen OA, Arepalli S, Assareh AA, Barral S, Bastin ME, Becker DM, Becker JT, Bennett DA, Blangero J, van Bokhoven H, Boomsma DI, Brodaty H, Brouwer RM, Brunner HG, Buckner RL, Buitelaar JK, Bulayeva KB, Cahn W, Calhoun VD, Cannon DM, Cavalleri GL, Cheng CY, Cichon S, Cookson MR, Corvin A, Crespo-Facorro B, Curran JE, Czisch M, Dale AM, Davies GE, de Craen AJM, de Geus EJC, de Jager PL, de Zubicaray GI, Deary IJ, Debette S, DeCarli C, Delanty N, Depondt C, DeStefano A, Dillman A, Djurovic S, Donohoe G, Drevets WC, Duggirala R, Dyer TD, Enzinger C, Erk S, Espeseth T, Fedko IO, Fernández G, Ferrucci L, Fisher SE, Fleischman DA, Ford I, Fornage M, Foroud TM, Fox PT, Francks C, Fukunaga M, Gibbs JR, Glahn DC, Gollub RL, Göring HHH, Green RC, Gruber O, Gudnason V, Guelfi S, Håberg AK, Hansell NK, Hardy J, Hartman CA, Hashimoto R, Hegenscheid K, Heinz A, le Hellard S, Hernandez DG, Heslenfeld DJ, Ho BC, Hoekstra PJ, Hoffmann W, Hofman A, Holsboer F, Homuth G, Hosten N, Hottenga JJ, Huentelman M, Pol HEH, Ikeda M, Jack Jr CR, Jenkinson M, Johnson R, Jönsson EG, Jukema JW, Kahn RS, Kanai R, Kloszewska I, Knopman DS, Kochunov P, Kwok JB, Lawrie SM, Lemaître H, Liu X, Longo DL, Lopez OL, Lovestone S, Martinez O, Martinot JL, Mattay VS, McDonald C, McIntosh AM, McMahon FJ, McMahon KL, Mecocci P, Melle I, Meyer-Lindenberg A, Mohnke S, Montgomery GW, Morris DW, Mosley TH, Mühleisen TW, Müller-Myhsok B, Nalls MA, Nauck M, Nichols TE, Niessen WJ, Nöthen MM, Nyberg L, Ohi K, Olvera RL, Ophoff RA, Pandolfo M, Paus T, Pausova Z, Penninx BWJH, Pike GB, Potkin SG, Psaty BM, Reppermund S, Rietschel M, Roffman JL, Romanczuk-Seiferth N, Rotter JI, Ryten M, Sacco RL, Sachdev PS, Saykin AJ, Schmidt R, Schmidt H, Schofield PR, Sigursson S, Simmons A, Singleton A, Sisodiya SM, Smith C, Smoller JW, Soininen H, Steen VM, Stott DJ, Sussmann JE, Thalamuthu A, Toga AW, Traynor BJ, Troncoso J, Tsolaki M, Tzourio C, Uitterlinden AG, Hernández MCV, van der Brug M, van der Lugt A, van der Wee NJA, van Haren NEM, van ’t Ent D, van Tol MJ, Vardarajan BN, Vellas B, Veltman DJ, Völzke H, Walter H, Wardlaw JM, Wassink TH, Weale ME, Weinberger DR, Weiner MW, Wen W, Westman E, White T, Wong TY, Wright CB, Zielke RH, Zonderman AB, Martin NG, van Duijn CM, Wright MJ, Longstreth WT, Schumann G, Grabe HJ, Franke B, Launer LJ, Medland SE, Seshadri S, Thompson PM, Ikram MA (2017). Novel genetic loci associated with hippocampal volume. Nat Commun.

[67] Vrijenhoek T, Buizer-Voskamp JE, van der Stelt I, Strengman E, Genetic R, Outcome in Psychosis C (2008). Recurrent CNVs disrupt three candidate genes in schizophrenia patients. Am J Hum Genet.

[68] Wang KS, Liu XF, Aragam N (2010). A genome-wide meta-analysis identifies novel loci associated with schizophrenia and bipolar disorder. Schizophr Res.

[69] Lesch KP, Timmesfeld N, Renner TJ, Halperin R, Roser C, Nguyen TT (2008). Molecular genetics of adult ADHD: converging evidence from genome-wide association and extended pedigree linkage studies. J Neural Transm (Vienna).

[70] Wang KS, Tonarelli S, Luo X, Wang L, Su B, Zuo L, Mao CX, Rubin L, Briones D, Xu C (2015). Polymorphisms within ASTN2 gene are associated with age at onset of Alzheimer’s disease. J Neural Transm (Vienna).

[71] Hafner A, Obermajer N, Kos J (2010). Gamma-1-syntrophin mediates trafficking of gamma-enolase towards the plasma membrane and enhances its neurotrophic activity. Neurosignals.

[72] Lencz T, Lambert C, DeRosse P, Burdick KE, Morgan TV, Kane JM, Kucherlapati R, Malhotra AK (2007). Runs of homozygosity reveal highly penetrant recessive loci in schizophrenia. Proc Natl Acad Sci U S A.

[73] Chen BJ, Mills JD, Takenaka K, Bliim N, Halliday GM, Janitz M (2016). Characterization of circular RNAs landscape in multiple system atrophy brain. J Neurochem.

[74] Rusina R, Bourdain F, Matej R (2007). Multiple system atrophy and Alzheimer’s disease: a case report of a rare association of two neuro-degenerative disorders. Rev Neurol (Paris).

[75] Chen H, Cade BE, Gleason KJ, Bjonnes AC, Stilp AM, Sofer T, Conomos MP, Ancoli-Israel S, Arens R, Azarbarzin A, Bell GI, Below JE, Chun S, Evans DS, Ewert R, Frazier-Wood AC, Gharib SA, Haba-Rubio J, Hagen EW, Heinzer R, Hillman DR, Johnson WC, Kutalik Z, Lane JM, Larkin EK, Lee SK, Liang J, Loredo JS, Mukherjee S, Palmer LJ, Papanicolaou GJ, Penzel T, Peppard PE, Post WS, Ramos AR, Rice K, Rotter JI, Sands SA, Shah NA, Shin C, Stone KL, Stubbe B, Sul JH, Tafti M, Taylor KD, Teumer A, Thornton TA, Tranah GJ, Wang C, Wang H, Warby SC, Wellman DA, Zee PC, Hanis CL, Laurie CC, Gottlieb DJ, Patel SR, Zhu X, Sunyaev SR, Saxena R, Lin X, Redline S (2018). Multiethnic meta-analysis identifies RAI1 as a possible obstructive sleep apnea-related quantitative trait locus in men. Am J Respir Cell Mol Biol.

[76] Emamian F, Khazaie H, Tahmasian M, Leschziner GD, Morrell MJ, Hsiung GY (2016). The association between obstructive sleep apnea and Alzheimer’s disease: a meta-analysis perspective. Front Aging Neurosci.

