# Supplementary material for: Targeting Neuroplasticity, Cardiovascular, and Cognitive-Associated Genomic Variants in Familial Alzheimer’s Disease
Source: Mol Neurobiol. 2018 Aug 15;56(5):3235–43. doi: 10.1007/s12035-018-1298-z (PMC6476862; doi:10.1007/s12035-018-1298-z)
Supplement: Supplementary file 1 — (DOCX 331 kb) [file 12035_2018_1298_MOESM1_ESM.docx]

**Supplementary Material for:**

**Targeting Neuroplasticity, Cardiovascular, and Cognitive-Associated Genomic Variants in Familial Alzheimer’s Disease**

Jorge I. Vélez^1,2,*^, Francisco Lopera^3*^, Penelope K. Creagh^1^, Laura B. Piñeros^4^, Debjani Das^5^,
Martha L. Cervantes-Henríquez^2,6^, Johan E. Acosta-López^6^, Mario A. Isaza – Ruget^7^, Lady G. Espinosa^7^, Simon Easteal^5^, Gustavo A. Quintero^8^, Claudia Tamar Silva^4^, Claudio A. Mastronardi^1,9,*,#^, Mauricio Arcos-Burgos^1,4,*,#^

July 20, 2018

^1^ *Genomics and Predictive Medicine Group, Department of Genome Sciences, John Curtin School of Medical Research, The Australian National University, Canberra, ACT, 2600, Australia.*

^2^ *Universidad del Norte, Barranquilla, Colombia.*

^3^ *Neuroscience Research Group, University of Antioquia, Medellín, Colombia.*

^4^ *GENIUROS, Center for Research in Genetics and Genomics, Institute of Translational Medicine, School of Medicine and Health Sciences, Universidad del Rosario, Bogotá, Colombia.*

^5^ *Genome Diversity and Health Group, Department of Genome Sciences, John Curtin School of Medical Research, The Australian National University, Canberra, ACT, 2600, Australia.*

^6^ *Grupo de Neurociencias del Caribe, Universidad Simón Bolívar, Barranquilla, Colombia.*

^7^*INPAC Research Group, Fundación Universitaria Sanitas,Bogotá, Colombia.*

^8^*Studies in Translational Microbiology and Emerging Diseases (MICROS) Research Group, School of Medicine and Health Sciences, Universidad del Rosario, Bogotá, Colombia.*

^9^*Neuroscience Group (NeUROS), Institute of Translational Medicine, School of Medicine and Health Sciences, Universidad del Rosario, Bogotá, Colombia.*

*^*^ These authors contributed equally to this work.*

*^#^ Correspondence to be directed to*

Mauricio Arcos-Burgos, MD, PhD

Director,

Institute of Translational Medicine,

School of Medicine and Health Sciences,

Universidad del Rosario,

Bogotá, Colombia.

E-mail: [oscarma.arcos@urosario.edu.co](mailto:oscarma.arcos@urosario.edu.co)

The authors have declared they have no financial interests to disclose.

**Supplementary Table 1.** Single nucleotide polymorphisms (SNPs) genotyped in this study

| **Chromosome** | **SNP*^a^*** | **Closest gene** | **Alleles** | **MAF** | **Association** | **Reference** |
| --- | --- | --- | --- | --- | --- | --- |
| 19 | rs3764650 | *ABCA7* | T/G | 0.11 | Alzheimer’s disease | Hollingworth et al. (2011) |
| 10 | rs1801253 | *ADRB1* | C/G | 0.31 | Blood Pressure | Johnson et al. (2011b); Johnson et al. (2011a) |
| 1 | rs2004776 | *AGT* | C/T | 0.26 | Blood Pressure | Johnson et al. (2011b); Johnson et al. (2011a) |
| X | rs12007229 | *AR* | C/A | 0.12 | Dementia | Schrijvers et al. (2012) |
| 11 | rs633185 | *ARHGAP42* | G/C | 0.3 | Blood Pressure | Bis et al. (2012) |
| 9 | rs7852872 | *ASTN2* | C/G | 0.39 | Neuroanatomy | Bis et al. (2012) |
| 1 | rs2942354 | *ATF3* | C/A | 0.44 | Neuroanatomy | Melville et al. (2012) |
| 12 | rs2681472 | *ATP2B1* | A/G | 0.12 | Blood Pressure | Levy et al. (2009) |
| 6 | rs805303 | *BAG6* | G/A | 0.31 | Blood Pressure | Bis et al. (2012) |
| 11 | rs6265 | *BDNF* | C/T | 0.2 | Cognition | Harris et al. (2006);  Mandelman and Grigorenko (2012) |
| 2 | rs744373 | *BIN1* | A/G | 0.31 | Alzheimer’s disease | Naj et al. (2011); Seshadri S and et al (2010) |
| 6 | rs9296559 | *CD2AP* | T/C | 0.27 | Alzheimer’s disease | Naj et al. (2011); Hollingworth et al. (2011) |
| 19 | rs34813869 | *CD33* | A/G | 0.29 | Alzheimer’s disease | Naj et al. (2011); Hollingworth et al. (2011) |
| 16 | rs5882 | *CETP* | G/A | 0.36 | Cognition | Izaks et al. (2012) |
| 20 | rs1044396 | *CHRNA4* | G/A | 0.42 | Neuroanatomy | Markett et al. (2013) |
| 8 | rs11136000 | *CLU* | C/T | 0.35 | Alzheimer’s disease | Naj et al. (2011); Lambert et al. (2009) |
| 22 | rs4680 | *COMT* | G/A | 0.48 | Cognition | Houlihan et al. (2009); Harris et al. (2005) |
| 1 | rs3818361 | *CR1* | G/A | 0.26 | Alzheimer’s disease | Naj et al. (2011); Lambert et al. (2009) |
| 15 | rs1378942 | *CSK* | A/C | 0.32 | Blood Pressure | Bis et al. (2012) |
| 20 | rs6125962 | *CTNNBL1* | T/C | 0.6 | Cognition | Papassotiropoulos et al. (2013) |
| 15 | rs700518 | *CYP19A1* | C/T | 0.42 | Neuroanatomy | Bayer et al. (2013) |
| 2 | rs6741949 | *DPP4* | G/C | 0.43 | Neuroanatomy | Bis et al. (2012) |
| 11 | rs6277 | *DRD2* | A/G | 0.47 | Neuroanatomy | Markett et al. (2013) |
| 7 | rs11767557 | *EPHA1-AS1* | T/C | 0.2 | Alzheimer’s disease | Naj et al. (2011); Hollingworth et al. (2011) |
| 1 | rs6703865 | *F5* | G/A | 0.4 | Neuroanatomy | Melville et al. (2012) |
| 4 | rs1458038 | *FGF5* | C/T | 0.27 | Blood Pressure | Bis et al. (2012) |
| 10 | rs17314229 | *FRMD4A* | C/T | 0.09 | Alzheimer’s disease | Lambert et al. (2013) |
| 10 | rs2446581 | *FRMD4A* | A/G |  | Alzheimer’s disease | Lambert et al. (2013) |
| 10 | rs7081208 | *FRMD4A* | G/A | 0.29 | Alzheimer’s disease | Lambert et al. (2013) |
| 16 | rs3751812 | *FTO* | G/T | 0.46 | Neuroanatomy | Ho et al. (2010) |
| 2 | rs2298948 | *GCFC2* | T/C | 0.33 | Neuroanatomy | Melville et al. (2012) |
| 20 | rs6015450 | *GNAS-EDN3* | A/G | 0.07 | Blood Pressure | Bis et al. (2012) |
| 12 | rs10845840 | *GRIN2B* | C/T | 0.46 | Neuroanatomy | Kohannim et al. (2012) |
| 6 | rs1799945 | *HFE* | C/G | 0.18 | Blood Pressure | Bis et al. (2012) |
| 12 | rs7294919 | *HRK* | T/C | 0.1 | Neuroanatomy | Bis et al. (2012) |
| 14 | rs4644 | *LGALS3* | C/A | 0.49 | Cognition | Trompet et al. (2012) |
| 13 | rs9315702 | *LHFP* | C/A | 0.43 | Neuroanatomy | Melville et al. (2012) |
| X | rs2239464 | *MECP2* | G/A | 0.22 | Neuroanatomy | Joyner et al. (2009) |
| 11 | rs12808148 | *MMP12* | T/C | 0.2 | Dementia | Kamboh et al. (2012) |
| 11 | rs4938933 | *MS4A4A* | T/C | 0.5 | Alzheimer’s disease | Naj et al. (2011) |
| 11 | rs670139 | *MS4A4E* | G/T | 0.34 | Alzheimer’s disease | Hollingworth et al. (2011) |
| 11 | rs610932 | *MS4A6A* | T/G | 0.45 | Alzheimer’s disease | Hollingworth et al. (2011) |
| 12 | rs17178006 | *MSRB3* | T/G | 0.09 | Neuroanatomy | Bis et al. (2012) |
| 6 | rs11754661 | *MTHFD1L* | G/A | 0.07 | Alzheimer’s disease | Beecham et al. (2009); Naj et al. (2011) |
| 1 | rs17367504 | *MTHFR* | A/G | 0.17 | Blood Pressure | Bis et al. (2012) |
| 7 | rs3918226 | *NOS3* | C/T | 0.04 | Blood Pressure | Salvi et al. (2012) |
| 5 | rs1173771 | *NPR3* | A/G | 0.49 | Blood Pressure | Bis et al. (2012) |
| 20 | rs4334545 | *NTSR1* | C/T | 0.29 | Neuroanatomy | Li et al. (2013) |
| 1 | rs678849 | *OPRD1* | C/T | 0.47 | Neuroanatomy | Roussotte et al. (2014) |
| 4 | rs11549976 | *PAICS* | A/C | 0.08 | Dementia | Velez et al. (2013) |
| 1 | rs1136410 | *PARP1* | A/G | 0.15 | Neuroanatomy | Nho et al. (2013) |
| 8 | rs10808746 | *PDE7A* | G/A | 0.48 | Cognition | De Jager et al. (2012) |
| 11 | rs3851179 | *PICALM* | C/T | 0.41 | Alzheimer’s disease | Naj et al. (2011); Harold et al. (2009) |
| 1 | rs3917836 | *SELP* | T/C | 0.05 | Neuroanatomy | Melville et al. (2012) |
| 8 | rs16914781 | *SNTG1* | A/G | 0.4 | Dementia | Velez et al. (2013) |
| 11 | rs668387 | *SORL1* | T/C | 0.48 | Alzheimer’s disease | Rogaeva et al. (2007) |
| 11 | rs2618516 | *SPON1* | C/T | 0.36 | Neuroanatomy | Jahanshad et al. (2013) |
| 11 | rs11023139 | *SPON1* | G/A | 0.06 | Cognition | Sherva et al. (2013) |
| 9 | rs11139399 | *TLE1* | T/C | 0.41 | Neuroanatomy | Melville et al. (2012) |
| 6 | rs1800629 | *TNF* | G/A | 0.17 | Neuroanatomy | Baune et al. (2012) |
| 17 | rs3744028 | *TRIM65* | T/C | 0.2 | Neuroanatomy | Fornage et al. (2011) |
| 5 | rs163030 | *WDR41* | A/C | 0.47 | Neuroanatomy | Stein et al. (2011) |
| 12 | rs6581612 | *WIF1* | C/A | 0.25 | Neuroanatomy | Bis et al. (2012) |
| 19 | rs3746319 | *ZNF224* | G/A | 0.19 | Dementia | Shulman et al. (2010) |

*^a^* UCSC GRCh37/hg19 coordinates. UCSC GRCh37/hg19 coordinates. Chr: Chromosome; MAF: Minor allele frequency; SNP: single

nucleotide polymorphisms.

**Power analysis**

We conducted a power analysis following the strategy outlined in Vélez et al. (2016) using the ‘pwr’ package (Champely, 2017) in R version 3.3.0 (R Core Team, 2017). In our first approach, assuming that all tested genetic markers were biallelic (that is, a a *k*=3 group design), 78 individuals would be sufficient to detect >85% true positives and a large effect (defined by the Cohen’s *f* parameter; *f*=3, Supplementary Figure 1) when *m=*64 genetic variants are tested for association. The selection of such effect is based on the observed effect size in our sample (see also the $\hat{\beta}$ coefficients in Table 1a of the main manuscript).

**Supplementary Figure 1.** (a) Sample size (*n*) as a function of the Cohen’s effect size (*d*) and power. The type I error probability used for calculations is 0.05/64. In (b) the red dot corresponds to *n=*78, power > 80% and a huge effect *f =*3. Cohen (1988) suggests that values of *f=*0.2, 0.5, and 0.8 represent small, medium, and large effect sizes.

In addition, we evaluated the power of a sample of *n*_1_ individuals with an ADAOO < 48 years (early-onset AD) and *n*_2_ individuals with ADAOO ≥ 48 years (early-onset AD), using a two-sample *t-*test, for a wide range of effect sizes (*f*), where *f* is calculated as $f=\left| \frac{\mu_{1}-\mu_{2}}{\sigma} \right|$. In our context, $\mu_{1}$ and $\mu_{2}$are the population ADAOO for individuals with early- and late-onset AD, respectively, and $\sigma^{2}$ is the common variance. We estimated $\mu_{1}$and $\mu_{2}$ with the correspondent sample means (i.e., 44.8 and 52.5, respectively) and $\sigma^{2}$as $\hat{\sigma}^{2}=\frac{S_{1}^{2}}{n_{1}}+\frac{S_{2}^{2}}{n_{2}}$, with $S_{1}^{2}$and $S_{2}^{2}$ the sample estimates of the variance for the group of individuals with early- and late-ADAOO, that is, 1.96^2^ and 3.89^2^, respectively. Thus, the sample effect size is *f=*11.17 (which corresponds to an extremely large effect size according to Cohen, 1998). The observed power of our study design is >99% when a type I error probability of 5/64 % is used. Now, assuming no changes in the variance estimates and a sample size of *n*=78 individuals, and a power of 99%, the minimum detectable effect size is *f=*0.989. Hence, our study design has a power of >95% to detect large sizes and hence the ADAOO can be safely tested with our sample size.

**REFERENCES**

1. Baune, B.T., Konrad, C., Grotegerd, D., et al. 2012. Tumor necrosis factor gene variation predicts hippocampus volume in healthy individuals. Biological psychiatry 72, 655–662.
2. Bayer, J., Rune, G., Kutsche, K., et al. 2013. Estrogen and the male hippocampus: Genetic variation in the aromatase gene predicting serum estrogen is associated with hippocampal gray matter volume in men. Hippocampus 23, 117–121.
3. Beecham, G.W., Martin, E.R., Li, Y.J., et al. 2009. Genome-wide Association Study Implicates a Chromosome 12 Risk Locus for Late-Onset Alzheimer Disease. American journal of human genetics 84, 35–43.
4. Bis, J.C., DeCarli, C., Smith, A.V., et al. 2012. Common variants at 12q14 and 12q24 are associated with hippocampal volume. Nat Genet 44, 545–551.
5. Champely, S. 2017. pwr: Basic Functions for Power Analysis. R package version 1.2-1. <https://CRAN.R-project.org/package=pwr>
6. Cohen J. Statistical power analysis for the behavioral sciences. 2nd ed. ed: Hillsdale, NJ: Lawrence Erlbaum; 1998
7. De Jager, P.L., Shulman, J.M., Chibnik, L.B., et al. 2012. A genome-wide scan for common variants affecting the rate of age- related cognitive decline. Neurobiology of aging 33, 1017.e1–1017.e15.
8. Fornage, M., Debette, S., Bis, J.C., et al. 2011. Genome- wide association studies of cerebral white matter lesion burden. Annals of Neurology 69, 928–939.
9. Gottesman, I., Gould, T., 2003. The Endophenotype concept in psychiatry: Etymology and strategic intentions. The American Journal of Psychiatry 160, 636–645.
10. Harold, D., Abraham, R., Hollingworth, P., et al. 2009. Genome-wide association study identifies variants at CLU and PICALM associated with Alzheimer’s disease. Nat Genet 41, 1088–1093.
11. Harris, S.E., Wright, A.F., Hayward, C., Starr, J.M., Whalley, L.J., Deary, I.J., 2005. The functional COMT polymorphism, Val 158 Met, is associated with logical memory and the personality trait intellect/imagination in a cohort of healthy 79 year olds. Neuroscience Letters 385, 1–6.
12. Harris, S.E., Fox, H., Wright, A.F., Hayward, C., Starr, J.M., Whalley, L.J., Deary, I.J., 2006. The brain-derived neurotrophic factor Val66Met polymorphism is associated with age-related change in reasoning skills. Molecular Psychiatry 11, 505–513.
13. Ho, A.J., Stein, J.L., Hua, X., et al. 2010. A commonly carried allele of the obesity- related FTO gene is associated with reduced brain volume in the healthy elderly. Proceed- ings of the National Academy of Sciences 107, 8404–8409.
14. Hollingworth, P., Harold, D., Sims, R., et al. 2011.Common variants at ABCA7, MS4A6A/MS4A4E, EPHA1, CD33 and CD2AP are associated with Alzheimer’s disease. Nat Genet 43, 429–435.
15. Houlihan, L.M., Harris, S.E., Luciano, M., Gow, A.J., Starr, J.M., Visscher, P.M., Deary, I.J., 2009. Replication study of candidate genes for cognitive abilities: the Lothian Birth Cohort 1936. Genes, Brain and Behavior 8, 238–247.
16. Izaks, G.J., van der Knaap, A.M., Gansevoort, R.T., Navis, G., Slaets, J.P.J., Dullaart, R.P.F., 2012. Cholesteryl Ester Transfer Protein (CETP) genotype and cognitive function in persons aged 35 years or older. Neurobiology of aging 33, 1851.e7–1851.e16.
17. Jahanshad, N., Rajagopalan, P., Hua, X., et al. 2013. Genome-wide scan of healthy human connectome discovers SPON1 gene variant influencing dementia severity. Proceedings of the National Academy of Sciences 110, 4768–4773.
18. Johnson, A.D., Newton-Cheh, C., Chasman, D.I., et al. 2011a. Asso- ciation of hypertension drug target genes with blood pressure and hypertension in 86,588 individuals. Hypertension 57, 903–910.
19. Johnson, T., Gaunt, T.R., Newhouse, S.J., et al. 2011b. Blood pressure loci identified with a gene-centric array. American journal of human genetics 89, 688–700.
20. Joyner, A.H., J, C.R., Bloss, C.S., et al. 2009. A common MECP2 haplotype associates with reduced cortical surface area in humans in two independent populations. Proceedings of the National Academy of Sciences 106, 15483–15488.
21. Kamboh, M.I., Demirci, F.Y., Wang, X., et al. 2012. Genome-wide association study of Alzheimer’s disease. Transl Psychiatry 2, e117.
22. Kohannim, O., Hibar, D.P., Jahanshad, N., Stein, J.L., Hua, X., Toga, A.W., Jack, C.R.J., Weiner, M.W., Thompson, P.M., 2012. Predicting Temporal Lobe Volume on MRI from Genotypes using L^1^-L^2^ regularized regression. Proceedings / IEEE International Symposium on Biomedical Imaging: from nano to macro. IEEE International Symposium on Biomedical Imaging, 1160–1163.
23. Lambert, J.C., Heath, S., Even, G., et al. 2009. Genome-wide association study identifies variants at CLU and CR1 associated with Alzheimer’s disease. Nat Genet 41, 1094–1099.
24. Lambert, J.C., Grenier-Boley, B., Harold, D., et al. 2013. Genome-wide haplotype associa- tion study identifies the FRMD4A gene as a risk locus for Alzheimer’s disease. Molecular Psychiatry 18, 461–470.
25. Levy, D., Ehret, G.B., Rice, K., et al. 2009. Genome-wide association study of blood pressure and hypertension. Nat Genet 41, 677–687.
26. Li, J., Chen, C., Lei, X., Wang, Y., Chen, C., He, Q., Moyzis, R.K., Xue, G., Zhu, B., Cao, Z., Dong, Q., 2013. The NTSR1 gene modulates the association between hippocampal structure and working memory performance. NeuroImage 75, 79–86.
27. Mandelman, S.D., Grigorenko, E.L., 2012. BDNF Val66Met and cognition: all, none, or some? A meta-analysis of the genetic association. Genes, Brain and Behavior 11, 127–136.
28. Markett, S., Reuter, M., Montag, C., Weber, B., 2013. The dopamine D2 receptor gene DRD2 and the nicotinic acetylcholine receptor gene CHRNA4 interact on striatal gray matter volume: evidence from a genetic imaging study. NeuroImage 64, 167–172.
29. Melville, S.A., Buros, J., Parrado, A.R., Vardarajan, B., Logue, M.W., Shen, L., Risacher, S.L., Kim, S., Jun, G., DeCarli, C., Lunetta, K.L., Baldwin, C.T., Saykin, A.J., Farrer, L.A., the Alzheimer’s Disease Neuroimaging, I., 2012. Multiple loci influencing hippocampal degeneration identified by genome scan. Annals of Neurology 72, 65–75.
30. Naj, A.C., Jun, G., Beecham, G.W., et al. 2011. Common variants at MS4A4/MS4A6E, CD2AP, CD33 and EPHA1 are asso- ciated with late-onset Alzheimer’s disease. Nat Genet 43, 436–441.
31. Nho, K., Corneveaux, J.J., Kim, S., et al. 2013. Whole-exome sequencing and imaging genetics identify functional variants for rate of change in hippocampal volume in mild cognitive impairment. Molecular Psychiatry 18, 781–787.
32. Papassotiropoulos, A., Stefanova, E., Vogler, C., et al. 2013. A genome-wide survey and functional brain imaging study identify CTNNBL1 as a memory-related gene. Molecular Psychiatry 18, 255–263.
33. R Core Team (2017). R: A language and environment for statistical computing. R Foundation for Statistical Computing, Vienna, Austria. URL <https://www.R-project.org/>
34. Rogaeva, E., Meng, Y., Lee, J.H., et al. 2007. The neuronal sortilin-related receptor SORL1 is genetically associated with Alzheimer disease. Nat Genet 39, 168–177.
35. Roussotte, F.F., Jahanshad, N., Hibar, D.P., et al. 2014. A commonly carried genetic variant in the delta opioid receptor gene, OPRD1, is associated with smaller regional brain volumes: replication in elderly and young populations. Human brain mapping 35, 1226–1236.
36. Salvi, E., Kutalik, Z., Glorioso, N., et al. 2012. Genomewide Association Study Using a High-Density Single Nucleotide Polymorphism Array and Case-Control Design Identifies a Novel Essential Hypertension Susceptibility Locus in the Promoter Region of Endothelial NO Synthase. Hypertension 59, 248–255.
37. Schrijvers, E.M.C., Schurmann, B., Koudstaal, P.J., et al. 2012. Genome-Wide Association Study of Vascular Dementia. Stroke 43, 315–319.
38. Seshadri S, F.A.L.I.M., et al, 2010. Genome-wide analysis of genetic loci associated with Alzheimer Disease. JAMA 303, 1832–1840.
39. Sherva, R., Tripodis, Y., Bennett, D.A., Chibnik, L.B., Crane, P.K., De Jager, P.L., Farrer, L.A., Saykin, A.J., Shulman, J.M., Green, R.C., 2013. Genome-wide association study of the rate of cognitive decline in Alzheimer’s disease. Alzheimer’s & Dementia 10(1):45-52.
40. Shulman, J.M., Chibnik, L.B., Aubin, C., Schneider, J.A., Bennett, D.A., De Jager, P.L., 2010. Intermediate Phenotypes Identify Divergent Pathways to Alzheimer’s Disease. PLoS ONE 5, e11244.
41. Stein, J.L., Hibar, D.P., Madsen, S.K., Khamis, M., et al. 2011. Discovery and replication of dopamine- related gene effects on caudate volume in young and elderly populations (N=1198) using genome-wide search. Molecular Psychiatry 16, 927–937.
42. Trompet, S., Jukema, W., Mooijaart, S.P., Ford, I., Stott, D.J., Westendorp, R.G.J., de Craen, A.J.M., 2012. Genetic variation in galectin-3 gene associates with cognitive function at old age. Neurobiology of Aging 33, 2232.e1–2232.e9.
43. Vélez, J.I., Chandrasekharappa, S.C., Henao, E., et al. 2013. Pooling/bootstrap-based GWAS (pbGWAS) identifies new loci modifying the age of onset in PSEN1 p.Glu280Ala Alzheimer's disease. Molecular Psychiatry 18(5):568-75.
44. Vélez, J.I., Lopera, F., Sepulveda-Falla, D. et al. 2016. APOE*E2 allele delays age of onset in *PSEN1* E280A Alzheimer's disease. Mol Psychiatry 21(7):916-24
